# Optimising Procyclidine Prescribing and Monitoring in Patients on Long-Acting Injectable Antipsychotics: A Quality Improvement Project

**DOI:** 10.1192/bjo.2026.11383

**Published:** 2026-06-30

**Authors:** Prabin Gautam, Shalina Mitchell, Lola Osoba

**Affiliations:** Kent and Medway Mental Health NHS Trust, Dartford, United Kingdom

## Abstract

**Aims:**

Procyclidine is frequently prescribed to manage extrapyramidal side effects (EPSE) associated with Long-Acting Injectable (LAI) antipsychotics. However, long-term use carries risks of anticholinergic burden and potential for misuse. Following a Serious Untoward Incident (SUI) involving a fatal procyclidine overdose, this quality improvement project aimed to assess and improve adherence to Kent and Medway Mental Health NHS Trust (KMMH) guidelines within the Dartford, Gravesham, and Swanley Community Mental Health Team (DGS CMHT). The primary hypothesis was that structured review prompts would increase the frequency of attempted dose reductions and withdrawal.

**Methods:**

A retrospective clinical audit was conducted in two cycles using the Glasgow Antipsychotic Side-effect Scale (GASS) to monitor side effects. Cycle 1 (September 2023–January 2024) reviewed 36 patients on LAIs. Standards included: adherence to starting dose (2.5mg), regular reviews (3–4 monthly), and attempted withdrawal after 3 months of use. Following Cycle 1, interventions included team-wide dissemination of findings and the implementation of standardised prompts to encourage dose-reduction discussions during reviews. Cycle 2 (January 2025–December 2025) involved a re-audit of the caseload (n=100) to evaluate changes in prescribing and monitoring practices.

**Results:**

Cycle 1 identified 14/36 (39%) patients on regular procyclidine. Although 93% had recorded reviews, clinical inertia was evident: 0% adhered to the recommended BNF starting regimen, and no withdrawal plans were documented.

Cycle 2 (n=100 randomly selected patients on LAI) identified 18 patients (18%) currently prescribed procyclidine. Detailed analysis of these 18 cases revealed a shift toward active management. Successful dose reductions were achieved in 3/18 (17%) patients. Discussions regarding dose reduction were documented in a further 4/18 (22%) cases. Only 1/18 (6%) required a dose increase. Consequently, 44% of the procyclidine cohort in Cycle 2 received active interventions or discussions regarding de-prescribing.

**Conclusion:**

This project demonstrated that there was significant improvement in documentation by clinicians prescribing procyclidine. There was also an observed procyclidine prevalence of 18% in the current cohort as compared to 39% from the previous cycle.

